# Complementary Mechanisms Potentially Involved in the Pathology of Zika Virus

**DOI:** 10.3389/fimmu.2018.02340

**Published:** 2018-10-15

**Authors:** Chet Raj Ojha, Myosotys Rodriguez, Jessica Lapierre, Mohan Kumar Muthu Karuppan, Heather Branscome, Fatah Kashanchi, Nazira El-Hage

**Affiliations:** ^1^Department of Immunology, Herbert Wertheim College of Medicine, Florida International University, Miami, FL, United States; ^2^Laboratory of Molecular Virology, School of Systems Biology, George Mason University, Manassas, VA, United States

**Keywords:** Zika virus, toll-like receptor, autophagy, apoptosis, unfolded protein response

## Abstract

Zika virus (ZIKV) has emerged as a global health threat due to its neuro-teratogenic effect and wide range of transmission routes. Most recently, ZIKV infection has been linked with both autoimmune disorders in adults and neurodevelopmental disorders in newborns. Researchers are exploring potential cellular and molecular mechanisms underlying the neuro-teratogenicity and related consequences by using various *in vitro* cell culture methods and *in vivo* animal models. Though some of the putative viral entry receptors have been identified for ZIKV entry into the target cells, the exact mechanism of ZIKV entry or induced pathology are still not clear. Some of the important host cellular pathways including the toll-like receptor (TLR), autophagy, apoptosis and unfolded protein response (UPR) pathways are considered potential mechanism(s) for ZIKV induced neuroinflammation and for neurodevelopmental disorders. Since there is still a dire need for efficient treatment and vaccine to prevent ZIKV mediated disorders, a better understanding of the interaction between virus and host cellular pathways could pave the way for development of targeted therapeutic intervention. In this review, we are focusing on the recent advances and current knowledge regarding the interaction of ZIKV with abovementioned pathways so as to provide basic understanding to execute further research that could aid in the development of novel therapeutic strategy.

## Introduction

Zika virus (ZIKV) is an emerging infectious virus with significant public health threat because of its recent association with microcephaly, a neurodevelopmental and neurodegenerative disorder in newborns and Guillain barre syndrome (GBS), an autoimmune disorder related to muscle paralysis (1, 2). In terms of historic time-line, the virus was first isolated in 1947 from monkey host in the Zika forest of Uganda and few years later in 1952, neutralizing antibody against the virus was isolated from human sera (3, 4). The first major human outbreak of ZIKV infection was reported in Yap Island of Micronesia in 2007 followed by a major public health concern from outbreaks in French Polynesia in 2013 and most recently in Brazil, where the infection with ZIKV was linked to increased prevalence of Guillain Barre syndrome and microcephaly, respectively (1, 2). In a case study of French Polynesians diagnosed with GBS, infection by ZIKV was supported in ninety-eight percent (41 out of 42) of patients by the detection of anti-ZIKV-IgM or anti-ZIKV-IgG antibodies. Every individual in that study with GBS had neutralizing antibodies against ZIKV, as compared to 56% detected in the control population (5). In Colombia, 42% of people who tested positive for ZIKV were diagnosed with GBS; reflecting the increase in cases of this disease with the ZIKV outbreak in 2015-2017 (6). In July 2015, the Brazilian Department of Health announced a direct association of ZIKV with GBS and 3 months later, ZIKV was reported to be the main cause related to the congenital abnormality detected in microcephalic newborns. A retrospective analysis of ZIKV outbreak in French Polynesia further provided the evidence for maternal ZIKV infection in the first trimester of pregnancy as the risk factor for microcephaly in fetuses (7). A great deal of attention was placed on the spread of and protection against the *Aedes mosquitos*, as they were the predominant species of mosquitos transmitting the virus. Sexual transmission, albeit not significant, is also a possible route of transmitting the virus (8, 9).

Similar to other Flaviviruses, the ZIKV genome is about 11 kb long and contains a single open reading frame (ORF) flanked by noncoding regions on both 5′ and 3′ sides (10, 11). The ORF encodes a polyprotein of 3419 amino acids that is cleaved by both host and viral proteases post-translationally into the capsid (C), precursor of membrane (prM), envelope (E) and 7 nonstructural proteins (NS1, NS2A, NS2B, NS3, NS4A NS4B, and NS5) (12). In addition to controlling viral transcription and replication, the NS proteins are involved in attenuating host antiviral responses (13–15), while the envelope (E) protein mediates cellular attachment, entry, and fusion and is the major target for neutralizing antibodies (16, 17). Once the virus has made its way inside the host, an integrated defense network comprising of innate and adaptive immune responses works together to thwart viral infections (18). Viruses and their components are sensed by the host cell via different pattern recognition receptors (PRR) including the Toll-like receptors (TLRs) and the Retinoic acid inducible gene (RIG)-1 like receptors (RLRs) to initiate a cellular defense (19). The interferon (IFN) signaling pathway, unfolded protein response (UPR), DNA repair mechanism, autophagy and apoptosis are major cellular mechanistic defenses that can suppress viral replication and salvage the infected host cell (20–23). While it is still unclear how ZIKV elicits its pathogenicity related to neurodegeneration and neurodevelopmental disorders, studies have unraveled several potential avenues that may be involved in ZIKV pathogenesis. In this review, we attempt to compile the findings of recent studies to provide a current view on the molecular mechanisms associated with ZIKV infection.

## Spatiotemporal variants of ZIKV and how they translate to pathogenesis

Genetic and phylogenetic studies have revealed that ZIKV has evolved from 3 distinct lineages including the West African (Nigerian cluster), the East African (MR766 prototype cluster), and the Asian lineage. Genetic studies on the NS5 gene indicate that ZIKV was most likely originated from East Africa and spread toward West Africa and Asia approximately 50–100 years ago (24, 25). The African lineage viruses were sporadically associated with human infection over the past century whereas the Asian lineages have emerged as a new public health burden because of their capacity of transmitting from human to human and causing neurological abnormalities (26). Genetic changes among the ZIKV lineages are attributed to the global spread of new phenotypes and the emergence of increased neuro-pathogenicity. Phylogenetic and amino acid variant analysis of the spatiotemporally different strains of ZIKV; MR766 (Uganda 1947), FSS13025 (Cambodia 2010), P6-740 (Malaysia 1966), PRVABC59 (Puerto Rico 2015), and DAKAR 41519 (Senegal 1984); revealed their distinct lineages and significant amino acid differences in the viral polyprotein. Higher levels of accumulated changes were reported in the envelope protein, the terminal region of prM, the NS2A and the NS5 protein regions of ZIKV genomes (26).

Lineage specific differences in ZIKV infectivity, virulence and pathologies have been reported using *in vivo* mouse and *in vitro* cell culture model system. Studies in which either the signal transducer and activator of transcription-2 knock out (*Stat2*^−/−^) or the interferon alpha/beta receptor 1 knock out (*Ifnar1*^−/−^) mouse model were used, showed that the Asian strains including the Puerto-Rican, Malaysian and Cambodian strains of ZIKV caused delayed onset of disease, less severe disease phenotypes and lower lethality rates when compared to the African strains (MR766) of ZIKV (26). However, others have reported that both the Asian strain (H/PF/2013) and the African strain (Dakar 41519) have similar virulence and caused 100% lethality in the *Ifnar*^−/−^ mouse model in a different study (27). The inconsistency between the studies in regards to the Asian strains might be related to the inherent differences within specific lineages, differences in their amino acid sequences and the study design including the viral inoculation dose. Based on published data this suggests that among the Asian strains, the French Polynesian strain (H/PF/2013) is more lethal compared to the viral strains within the Asian lineage of ZIKV. Nonetheless, these studies have revealed the differences in pathogenesis and lethality associated with spatiotemporally different ZIKV lineages or strains. *In vitro* studies have also reported similar data showing a higher infection rate in neural stem cells (NSCs) and astrocytes with the African lineage ArB41644 as compared to the Asian lineage PRVABC59 and H/PF/2013. In addition, NSCs infected with ArB41644 showed higher levels of cell cycle impairment and antiviral response when compared to cells infected with PRVABC59 and H/PF/2013. The same study also reported a significant up-regulation in the genes encoding for DExD/H-Box helicase 58 (*DDX58*), interferon induced with helicase C domain 1 (*IFIH1*), toll-like receptor-3 (*TLR*3), and interferon beta 1 (*IFNB1*) and downregulation of C-X-C motif chemokine ligand 8 (*CXCL8*) in NSCs infected with African strains, while significant down-regulation in the genes encoding for the C-X-C Motif chemokine ligand 10 (CXCL10), caspase 1 (*CASP1*) and cathepsin S (*CTSS*) was reported in NSCs infected with Asian strains (28). These findings further suggest that the molecular mechanism underlying ZIKV infection and pathogenesis might be lineage specific and a single therapeutic approach targeting the African lineage may not necessarily be effective against the Asian lineage of ZIKV.

## ZIKV entry receptors

**Figure 1:**
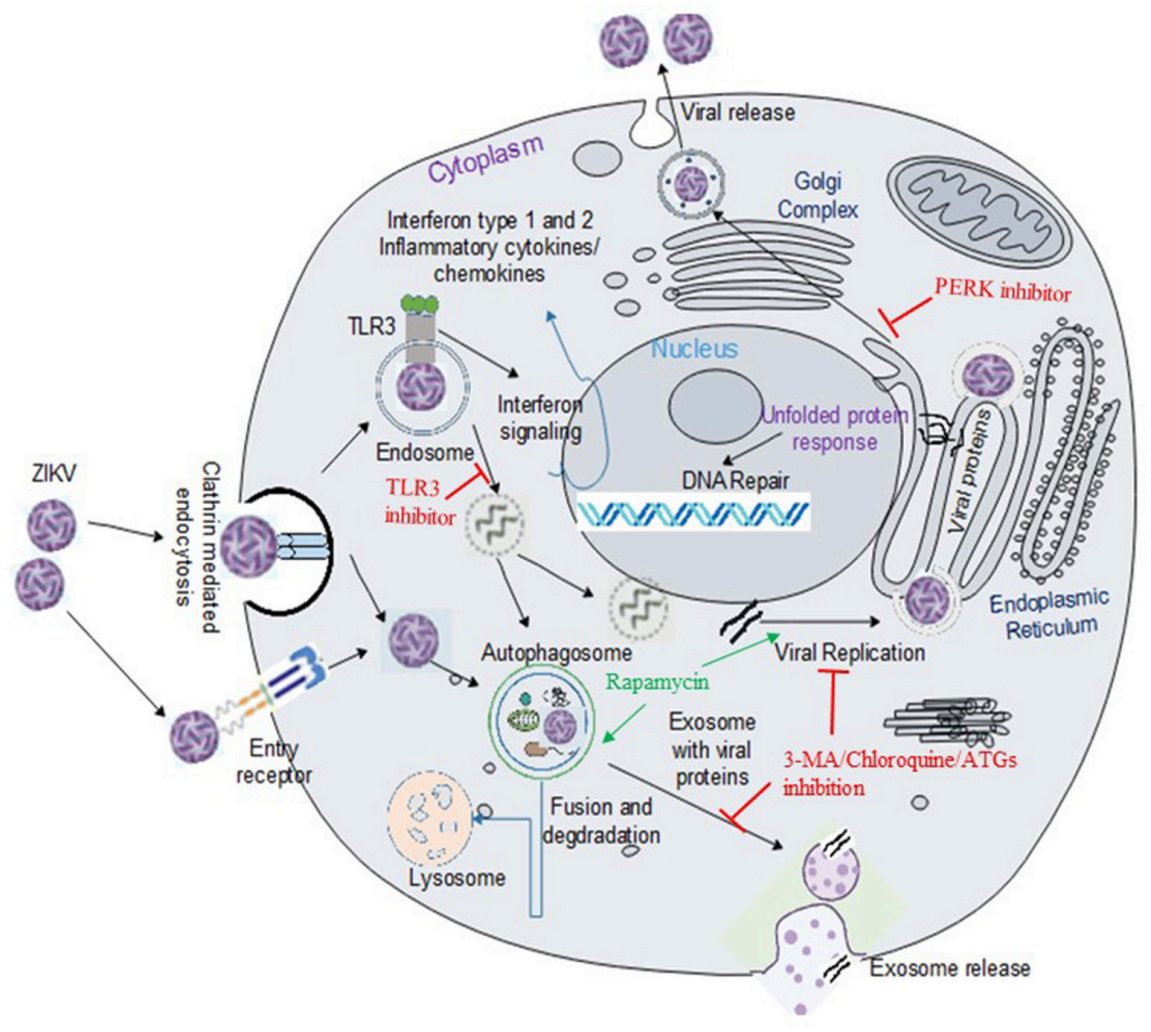
ZIKV entry, replication and interaction with cellular pathways in target cell. ZIKV enters the cell either via clathrin-mediated or receptor-mediated endocytosis. The acidic environment in the endosome induces viral fusion releasing genomic RNA. Viral RNA is subsequently translated into a polyprotein which is further processed by host and viral-encoded proteins. Flavivirus replication complex is assembled in close vicinity to endoplasmic reticulum (ER) membrane. Following viral replication, packaging occurs on the surface of the ER and the resultant immature virions are translocated to the Golgi complex where furin-mediated cleavage of prM to M generates mature virus that are released via exocytosis. ZIKV activates TLR3 (20) and the autophagy pathway (22), which may potentially mediate viral replication and survival within the cells. Exosomes released from infected cells have been reported to contain ZIKV proteins including NS1 (29). Flaviviruses manipulate the unfolded protein response (UPR) pathways in host by activation of one or more arms of the UPR which may lead to DNA repair, cell homeostasis or apoptosis (30). Green arrow indicates upregulation of the step in the pathway and red line indicates inhibition of the step in the pathway or viral replication.

ZIKV, similar to other members of the Flavivirus, enters the cell presumably by receptor mediated endocytosis (Figure 1) (31, 32). Several cell surface receptors including the dendritic-cell-specific ICAM-grabbing non-integrin (DC-SIGN), glucose-regulating protein 78 (GRP78/BiP) and Cluster of differential (CD)-14 associated molecules are believed to be the primary Flaviviral entry receptors (16, 31). Some low affinity molecules such as heparin and other glycosaminoglycan are also suggested to contribute to Flaviviral entry (33). However, not much is known about the mechanisms of viral binding and the interaction between putative receptors and co-receptors (16). Recently, the expression of DC-SIGN, Axl receptor tyrosine kinase (AXL) and tyrosine protein kinase receptor 3 (Tyro3) has been strongly correlated with ZIKV infection in human embryonic kidney (HEK)-293 cells while T cell immunoglobulin mucin (TIM)-1 or TIM-4 showed modest effects on ZIKV entry in HEK293 and A549 cells (23). Out of the putative ZIKV receptors, AXL is the most studied receptor in regards to ZIKV infection. AXL is a member of TAM (Tyro3-AXL-MER) family of receptors that was originally cloned from cancer cells (34). The receptor has two ligands including the growth arrest specific-6 (Gas6) and the protein S (35). AXL is involved in the innate immune response as well as other cellular mechanisms including proliferation, migration and aggregation in different cell types (36, 37). Exposure to neutralizing antibody against AXL or by gene silencing using small interfering RNA targeting the *AXL* gene led to a decrease in viral replication and infection, suggesting a role of AXL as a putative ZIKV entry receptor in human fibroblast cells (23). Similarly, Meertens L. et al. showed that human microglia and astrocytes isolated from developing brain expresses AXL receptors, which acts via the ligand growth arrest specific 6 (Gas6) to allow ZIKV entry and dampen innate immunity. Inhibition of AXL by synthetic decoy receptor (MYD1) and AXL kinase inhibitor (R428) resulted in the inhibition of ZIKV infection in the glial cells (32). Interestingly, it has recently been demonstrated that silencing the *AXL* gene was unable to inhibit viral entry but rather facilitates the upregulation of type 1 interferon signaling, indicating that AXL promotes ZIKV infection in glial cells by antagonizing type I interferon (IFN) signaling. Though many researchers are focusing on AXL as a putative ZIKV entry receptor and developing therapeutic target, studies have revealed that ZIKV entry and subsequent replication are not solely dependent on the AXL receptor (38–40), limiting the possibility of AXL and other TAM (Tyro, AXL, and Mer) receptors for therapeutic targeting against ZIKV infection.

## ZIKV pathogenesis and host immune response

Before the recent outbreaks, ZIKV infection was considered an asymptomatic or mild self-limiting febrile illness resolving within a few days. Wide range of tissue tropism, multiple routes of transmission and association with severe disease involving multi-organs are reported in the recent ZIKV outbreaks (41). Though ZIKV is thought to be less neurotropic than the other neurotropic Flaviviruses, the alarming consequence of ZIKV is its tropism for brain cells particularly neural progenitor cells leading to impairment of brain growth that may result in microcephaly and other neurological disorders (41). ZIKV is capable of infecting almost all cell types in the brain including astrocytes, microglia (32, 42), brain microvascular endothelial cells (43), pericytes (44), oligodendrocytes, and neurons (45). ZIKV can also infect eye including cornea and optic nerves causing uveitis and even blindness (46). Human to human transmission via sexual contact, body fluids and blood transfusion is the unique feature of ZIKV compared to other Flaviviruses, and might be the factor enhancing the stability of ZIKV in different body fluids and the broad viral tropism [reviewed in (47)]. High viral titer in the placenta suggests possible vertical transmission while presence of virus in testis, vagina and uterus supports the sexual transmission (47).

ZIKV is reported to modulate both the innate and adaptive immune-branches of the host defense mechanism; however, both branches may not be required to prevent the disease (48). Innate immune response is the primary host response that controls ZIKV infection and related pathogenesis. Type 1 IFN which includes the isoforms of IFN-α, IFN-β and other minor classes (such as IFN-ε, IFN-k, IFN-ω) are crucial for antiviral response and are produced by almost all cells in the body (49). The expression of type I IFN is regulated by an intracellular signaling pathway triggered by recognition of specific viral components such as viral double stranded (ds) or single stranded (ss) RNA or the replication intermediate of viral RNA by germline-encoded PRRs (19). ZIKV induces TLRs (mainly TLR3), RIG-1, melanoma differentiation associated protein 5 (MDA5), the interferon stimulated genes (ISGs) such as OAS2, ISG15, and MX1 along with IFN-β in various cell type (23). The primary role of innate immune response in controlling ZIKV infection is further supported by the finding that immuno-compromised individuals or animal models are more susceptible to ZIKV infection as well as the progression to overt disease (50). *Ifnar1*^−/−^ mice are susceptible to ZIKV infection up to 6 months of age and even the younger mice within the age of 3 weeks can succumb to overt illness (51).

However, in type 1 IFN deficient animal models, the adaptive immune response becomes critical (48, 50). ZIKV specific antibodies are reported to play a crucial role in controlling viral replication in the mouse model. Anti-ZIKV antibodies with variable neutralizing potency have been reported from the ZIKV infected patients. Interestingly, high neutralizing responses to ZIKV were associated with pre-existing Dengue virus (DENV) reactivity, suggesting possible cross-neutralization among the members of flavivirus (52). The envelope (E), pre-membrane (prM) and NS1 proteins are the major targets for ZIKV specific antibodies and therefore, are the attractive components for DNA vaccine (53). ZIKV-induced T-cell response was studied in immunocompetent C57BL/6 mice by tracking surrogate markers expressed by these cells. ZIKV sensitized CD4^+^ T cells polarized to a Th1 phenotype while CD8^+^ T cells differentiated into the activated effector phenotype, leading to the production of cytokines and cytolytic molecules (54). This novel ZIKV CD8^+^ T cell epitope identified for the envelope protein and recognized by many cells can pave the way for the design of tetramers to study epitope-specific T cell responses and development of ZIKV vaccine strategies (54). Depletions of T cells resulted in a decrease in body weight in ZIKV infected mice (48). Interestingly, pregnant C57BL/6 mice are reported to be more responsive to ZIKV-induced diminutions of cell-mediated immunity, leading to more viral replication and possible spread of virus from mother to fetus (48).

In order for ZIKV to evade and antagonize the host immune responses, the virus may have evolved multiple mechanisms. The nonstructural (NS) proteins, for example, can act as an immune response antagonist. First, ZIKV NS5 protein targets the proteasomal degradation of STAT2, leading to inhibition of type I IFN signaling (55). The viral proteins, NS1 and NS4B inhibit RLR-induced IFN-β activation at the Tank-binding kinase 1 (TBK1) level, a multifunctional protein implicated not only in the innate immunity but also in apoptosis and cell proliferation. Activation of STAT6 transcription factor and Interferon regulatory factor (IRF)-3 are mediated by TBK1 (48). ZIKV infection disrupts TBK1 relocation from the centrosome to the mitochondria which may lead to impaired mitosis and cell division (56). This suggests that TBK1 is one of the major targets of ZIKV infection (48). The other viral protein NS2B in conjunction with NS3 promotes the degradation of Janus kinase (JAK1) and the inhibition of the JAK-STAT signaling, followed by inhibition of viral-induced apoptosis and increased viral replication. Finally, NS1 and NS4B inhibit the expression of Type 1 IFN and consequently the degradation of NS2B and NS3 by the autophagy pathway with subsequent suppression of viral replication by this pathway. These findings suggest that the non-structural proteins act synergistically to restrict host antiviral responses and facilitate viral replication (57).

## ZIKV and the toll-like receptor-3 (TLR3) signaling pathway

TLR3 signaling pathway is a part of innate immune response and creates an antiviral state in viral infected cell. Normally, activation of TLR3 leads to the downstream signaling pathway which involves the recruitment of TIR domain containing adaptor inducing IFN-β (TRIF) and subsequent activation of transcription factors such as interferon regulatory factors (IRF)-3, IRF7 and nuclear factor kappa B (NF-κB) ultimately inducing the production of type 1 and type 2 IFN, pro-inflammatory cytokines, chemokines and ISGs (58, 59). Though the role of TLR3 in ZIKV pathogenesis is still under investigation, findings with DENV and West Nile virus (WNV) are not consistent (60–62). TLR3 deficient mice (*TLR3*^−/−^) are reported to be resistant to infection with the WNV (60) and the influenza virus (63). Similarly, *TLR3*^−/−^ mice infected with WNV showed reduced viral load in the brain, low levels of inflammatory molecules and less prominent neuropathology when compared to wild type mice, further suggesting the potential role of TLR3 in viral entry into brain and neuropathology (60). Conversely, a protective role of TLR3 with WNV infection has also been reported shedding a doubt on the exact role of TLR3 in flavivirus pathogenesis (62). In a separate study, activation of TLR3 was reported to block DENV type 2 replication via induction of IFN-β in human hepatoma cell line (61).

It is consistently reported that TLR3 was activated by ZIKV possibly by sensing the replication intermediate of viral RNA (20, 23). TLR3 was shown to be activated by ZIKV in human organoid cells and in murine neurospheres, causing perturbation of 41 genes related to neurodevelopment and reducing the organoid volume as seen in clinical microcephaly (20). The reduction in the organoid volume was correlated with the viral titers, indicating that activated TLR3 enhances ZIKV replication. Inhibition of TLR3/dsRNA by competitive inhibitor (thiophenecarboxamidopropionate) resulted in reversal of these phenotypic effects, while treatment with TLR3 agonist (poly I:C) mimicked ZIKV infection in terms of brain organoid volume (20). It is paradoxical that a component of innate immune response enhances viral replication and related pathogenesis. A proposed mechanism for ZIKV induced microcephaly suggested that ZIKV infection leads to TLR3-mediated hyper activation of innate immune response, which in turn cause transcriptional deregulation of the genes related to neurogenesis resulting in impaired neurogenesis (64).

## ZIKV and the autophagy pathway

Autophagy mediates the lysosomal degradation of long-lived proteins, cellular organelles and intracellular pathogens (65). The pathway is induced by nutrient deprivation or stress and promotes energy conservation and cell survival during starvation by recycling and salvage of cellular nutrients. Autophagy is a complex process involving more than 30 autophagy-related (ATG) proteins [reviewed in (66)], crucial for cellular development and differentiation (67), innate and adaptive immunity (68, 69), and programmed cell death (type II) (70, 71). Autophagosome formation involves initiation, nucleation, and expansion of the isolation membrane (72), and begins with the formation of the phagophore assembly site (PAS), the origin of which is still unclear in mammals (73, 74). The autophagosome fuses with endocytic and lysosomal compartments to form auto-endosome or autolysosome for subsequent degradation (72). Many signaling molecules such as mammalian target of rapamycin complex 1 (mTORC1), AMP-activated protein kinase (AMPK), death associated protein kinase (DAPK), c-jun N terminal kinase (JNK), Akt or protein kinase B (PKB), Casein kinase 2 (CK2), Insulin like growth factor (IGF-1), DNA damage regulated autophagy modulator (DRAM), p53, Forkhead box O (FOXO) and reactive oxygen species (ROS) are involved in autophagy regulation at various points [reviewed in (66)].

Microtubule associated protein light chain-3 (LC3) and sequestrome-1 (SQSTM1, also called p62) are two important markers of autophagosome formation and its maturation (75, 76). During autophagosome formation, the cytosolic form of LC3 (LC3-I) conjugates with phosphatidylethanolamine to form LC3-II. The lipidated form (LC3-II) is recruited to the autophagosome and gets degraded upon fusion with the lysosome in autolysosome, along with other damaged cellular components (75). Induction of autophagy as determined by conversion of LC3-I to LC3-II and formation of LC3 punctate was recorded in fetal neural stem cells (fNSCs) infected with strains of ZIKV (MR766 and IbH30656) in the presence or absence of the lysosome inhibitor, bafilomycin A1 (Baf A1). p62/SQSTM1 is a ubiquitin binding protein serving as an adaptor for the autophagy pathway by facilitating the delivery of cargo to autophagosomes (76). A decrease in p62/SQSTM1 levels with ZIKV infection was reported in fNSCs, indicating that autophagy maturation and lysosomal fusion were most likely not impaired. On the other hand, inhibiting the autophagy pathway with pharmacological inhibitors 3-methyladenine (3-MA) or chloroquine in both fNSCs and HeLa cells or gene silencing targeting the autophagy related gene (ATG)-3 in ZIKV-infected mouse embryo fibroblast (MEF) cells resulted in a staggering decrease in viral replication. Similar results were reported in siRNA-mediated inhibition of ATG-5 in MEF cells and siRNA-mediated inhibition of ATG-3 and ATG-13 in fNSCs (22). While, induction of autophagy with rapamycin promoted ZIKV replication in both fNSCs and HeLa cells. These findings present sustainable evidence that ZIKV infection induces autophagy in fNSCs, which in turn leads to increased ZIKV replication and viral load (22). Transient or stable expression of the NS4A and/or NS4B proteins in HeLa and fNPCs significantly induced the autophagy pathway, potentially through inhibition of the Akt-mTOR signaling pathway (22).

Studies have also shown that autophagy-related proteins play a significant role in placental defense against pathogenic microorganisms (77, 78). Although the exact mechanisms by which ZIKV crosses the placental barrier is still not clear, could be speculated that placental transfer may be mediated by ZIKV-packaged exosomes in the endoplasmic reticulum (ER) of trophoblastic cells. This process is similar to secretory or unconventional autophagy, in which viral polypeptides that lack N-terminal peptides are unable to be secreted from the ER to the Golgi secretory pathway and are instead exported via secretory autophagy pathway (29). Exosomes derived from ZIKV-infected HEK293 cells show co-localization of the viral protein NS1 with the exosome markers CD63 and CD9, demonstrating that NS1 is present in the exosome. Exosomes derived from HEK293 cells transfected with NS1 plasmids further validated the presence of NS1 in exosomes. As with the vesicular stomatitis virus (VSV), placenta-specific microRNAs packaged in exosomes secreted from human syncytium may also induce the autophagy pathway and modulate ZIKV replication (79). LC3-II protein expression was significantly upregulated at 6 and 12 hours-post-infection with ZIKV in human cytotrophoblast cells and further enhanced with Baf A1 (78). Treatment with the pharmacological inhibitors of autophagy (3-MA, chloroquine, and Baf A1) resulted in a significant decrease in ZIKV replication. Reciprocal increase in viral replication was observed with the autophagy inducers, rapamycin and torin 1 (78). Mice deficient for the autophagy-related gene Atg16L1 infected with ZIKV showed improved fetal outcomes while treatment with hydroxychloroquine in pregnant dams caused reduced ZIKV vertical transmission and limited placental damage and fetal death (77). Overall evidence supports a crucial role of autophagy in ZIKV replication and vertical transmission and that this pathway can be targeted for therapeutic interventions against ZIKV.

## ZIKV and the apoptosis pathway

Apoptosis, also known as type I programmed cell death, is an evolutionarily conserved cellular defense mechanism which removes damaged, infected or excess of cells from the body (80, 81). Cellular fate is determined by interactions of members of the BCL-2 family proteins which are the gatekeeper for apoptosis. (82). Apoptosis involves the activation of cysteine proteases (referred to as caspases) that ultimately induce cellular destruction (80, 83). Significant upregulation of caspase-3, an important apoptosis effector protein was reported in primary neurons infected with the Brazilian stain of ZIKV, suggesting an induction of apoptosis as a result of viral infection. Presence of caspase-3 was more readily observed in bystander cells than in ZIKV-infected cells which might be due to the release of pro-apoptotic factors by infected cells (84, 85). The tumor suppressor protein p53 is also implicated in ZIKV-mediated apoptosis, as inhibition of p53 by either pharmacological inhibitors or Ser15 phosphorylation, limits ZIKV induced apoptosis in neural progenitors (21, 86). p53 activates transcription of several pro-apoptotic genes including *Bax, Noxa* and *Puma*, while suppressing anti-apoptotic genes such as *survivin* resulting in activation of the apoptosis pathway (87). Contrastingly, ZIKV (PRVABC59) was also reported to induce the cyclin-dependent kinase inhibitor p21, which is an apoptosis inhibitor (88). Overall, the role of apoptosis in ZIKV infection is still not clear.

## ZIKV and the endoplasmic reticulum (ER) pathways

The ER provides a membrane platform for the biogenesis of Flavivirus replication complexes. Flaviviral infection leads to rearrangement of the ER membrane to from an organelle-like structure which provides an environment for viral replication. Viral proteins including structural and nonstructural proteins accumulated in the ER membrane facilitate the rearrangement by changing protein and lipid content of the ER membrane (89, 90). The hydrophilic transmembrane viral proteins, NS4A and NS4B, are primarily involved in membrane remodeling (91, 92). Membrane remodeling results in the formation of the so-called vesicle pockets or double membrane vesicles (DMVs) that facilitates the assembly of newly synthesized viral components and complete the virion packaging before trafficking to the Golgi complex. Therefore, interactions between the ER and flavivirus play a key role in providing a replication platform and assembly site [Reviewed in (30)].

On the other hand, synthesis of excessive viral protein may lead to ER stress. The intracellular signaling pathway referred to as the unfolded protein response (UPR) pathway next comes into scene to alleviate the ER stress. UPR pathway is a homeostatic signaling pathway that is activated to counteract ER stress by transcriptional induction of genes, retardation of global protein synthesis and ER associated degradation (93, 94). UPR is induced via either of three arms or stress sensors; protein kinase RNA-like ER kinase (PERK), inositol-requiring protein 1α (IRE1α) and activating transcription factor 6 (ATF6) (94). UPR mitigates unfolded protein load by pro-survival mechanisms such as ER membrane expansion, decreased influx of proteins into ER, induction of transcription of key components of protein folding. If the stress is not under control, then UPR induces selective autophagic degradation of ER (ER-phagy) or apoptosis [reviewed in (94)]. ZIKV has been reported to induce UPR pathway (as shown by upregulation of UPR-related genes expression) in human fetal brain cortex, mouse embryonic brain and *in vitro* hNSCs. Interestingly, pharmacological inhibitor of PERK (GSK2656157) reverted the ZIKV mediated impairment of neurogenesis and microcephaly in mouse embryos. Similarly, ZIKV-associated neurogenic defects were rescued by inhibition of PERK, whereas ZIKV replication was reduced by IRE-1α inhibitor in cortical cells (95).

ER stress can also lead to formation of stress granules (SGs) by phosphorylation of the eukaryotic initiation factor 2α (eIF2α). SGs are dynamic cytoplasmic granules composed of cellular mRNAs and stalled pre-initiation complexes. SGs play an important role in maintaining RNA homeostasis under stress conditions (96). SGs restrict viral replication by hiding cellular translational machinery. However, some viruses can counteract the innate defense mechanism provided by SGs assembly (97, 98). Likewise, ZIKV has evolved similar strategy to suppress SGs assembly despite activating UPR and phosphorylating eIF2α leading to global translational arrest. ZIKV proteins NS3 and NS4A are involved in translational arrest, whereas, capsid, NS3, NS2B, and NS4A co-operatively suppress SGs formation (99, 100). However, the precise mechanism of ZIKV induced suppression of SGs formation is still unclear.

## ZIKV animal research model and challenges

So far several animal models have been proposed for ZIKV infection, including immunocompetent mice (C57BL/6), mice lacking the receptors for interferon (IFNAR), mice neutralized with the IFNAR antibodies, mice lacking IRF3/5/7, immunocompetent non-human primates and chicken embryos [Reviewed in (101) and (102)]. The most commonly employed mice models are the A129 strain which lacks the receptors for type 1 interferon and the AG129 strain which lacks the receptors for both type 1 and 2 IFN (27, 103, 104). Comparable results can be achieved when using C57BL/6 mice treated with IFNAR1 monoclonal antibody prior to and during viral infection or using *Irf3*^−/−^ *Irf5*^−/−^ *Irf7*^−/−^ triple knockout mice (27, 103). Despite many advances in using ZIKV-infected mouse model in establishing the cell tropism, viral replication kinetics, host immune response, teratogenicity, and clinical manifestation, limited progress toward vaccine development has been made (53, 102). Structural and immunological dissimilarities between human and mice placenta, resistance of immunocompetent mice to ZIKV replication, skepticism about the relevance of data generated from immune compromised or genetically modified mice, limited availability of non-human primates and associated high cost are some of the limitations that are currently manifesting with the use of animal models (101).

## Vaccine and therapy development approach for ZIKV

Multiple vaccine platforms and approaches are being employed for ZIKV including DNA vaccines, subunit vaccines, whole inactivated virus vaccines and vectored vaccines (105). The nucleoside-modified mRNA encoding the pre-membrane and envelope (prM-E) glycoproteins encapsulated in lipid nanoparticle (mRNA-LNP) and ZIKV prM/E sequence DNA vaccine demonstrated robust protection against ZIKV by eliciting strong neutralizing antibody responses both in mice and non-human primates (53, 106). Some DNA vaccines are currently entering phase I clinical trial or clinical evaluation (107, 108). Some of the current approaches under evaluation are targeting viral proteins including, the envelope protein to block cellular entry and fusion, the NS5 protein to inhibit its RNA-dependent RNA polymerase and methyltransferase activities and the NS3 protein to inhibit protease and helicase activity (109–111). Repurposing therapy including the use of sofosbuvir, kitasamycin, lovastatin, 6-azauridine, palonosetron, and 5-fluorouracil are some potential drugs under consideration for ZIKV treatment (112–114). Another approach for developing ZIKV treatment and vaccines is to target host cellular molecules that are associated with increased viral replication and viral induced inflammation. Many of the putative molecular targets identified includes AXL, TLR3, small inducible cytokine B10 (IP10), interferon stimulated genes, interleukin (IL)-6 and Eukaryotic translation initiation factor (eIF)-2A protein kinase (eIF2AK2) (115). Targeted immunotherapy designed to boost the natural defenses against the pathogens can also combat ZIKV infection and associated complications. Monoclonal antibody against various epitopes of ZIKV structural proteins, isolated from B lymphocytes of infected human or animals have shown potent neutralizing activity against ZIKV (116). Cytotoxic T lymphocytes (CD8^+^) from infected mice also offer protection against ZIKV and prevent pathogenesis of ZIKV infection (117). Therapeutic implication of interferons as the inhibitors of ZIKV replication is challenged by the finding that ZIKV inhibits interferon signaling by targeting human STAT2 (55, 118). The summary of the potential therapeutic targets based on cellular signaling pathway are presented in Table 1.

**Table 1:** Summary of the potential complementary therapeutic targets for ZIKV.

**Cellular pathways**	**Targets**	**Expected outcome**	**References**
ZIKV entry receptors	1. Genetic silencing of AXL	• Inhibits ZIKV infection in various cell types directly or indirectly	(23, 32, 119)
	2. TAM kinase inhibitor (R428)		
	3. Synthetic decoy receptor (MYD1)		
	4. AXL/GAS6 inhibitors (small molecules-RU-301 and 302)		
Immunotherapy	5. ZIKV specific monoclonal antibodies	• Neutralizes the virus • Prevents viral replication	(54, 120, 121)
	6. DNA vaccine expressing ZIKV proteins		
	7. T-cells expressing ZIKV specific epitopes		
TLR3 pathway	8. Pharmacological inhibitor of TLR3/dsRNA	• Reverts ZIKV-induced reduction in neurospheres size • Reduces of ZIKV replication	(20)
	9. Genetic silencing of TLR3 by siRNA or CRISPR/cas9		
Autophagy pathway	10. Pharmacological inhibition of autophagy I. 3-methyladeneine II. Chloroquine III. Bafilomycin 1	• Suppress ZIKV replication, restrict vertical transmission of ZIKV • Prevent ZIKV related adverse fetal outcomes.	(22, 77, 78)
	11. Genetic silencing of ATG genes such as ATG3, ATG13, ATG16L1 etc.		
Apoptosis pathway	12. Pharmacological inhibitor of p53 (SER15 phosphorylation)	• Suppress ZIKV-induced apoptosis	(87, 122)
	13. Caspase inhibitors		
	14. Bcl-2 enhancement		
	15. Peptidomimetics		
ER signaling pathway	16. PERK inhibitor	• Prevent ZIKV-mediated impairment in neurogenesis	(95, 115)
	17. IRE1α inhibitor	• Prevents ZIKV replication in brain cortical cells	
	18. eIF2α inhibitor	• Prevent Stress Granule formation and its exploitation by ZIKV	

*ZIKV, Zika Virus; AXL, Axl tyrosine kinase receptor; TLR3, toll-like receptor-3; dsRNA, double stranded RNA; siRNA, small interfering RNA; CRISPR, Clustered Regularly Interspaced Short Palindromic Repeats; ATG, autophagy related protein; PERK, protein kinase RNA-like ER kinase (PERK); IRE1α, inositol-requiring protein 1α and eIF2AK2; eukaryotic initiation factor 2α*.

## Conclusions and future perspectives

**Figure 2:**
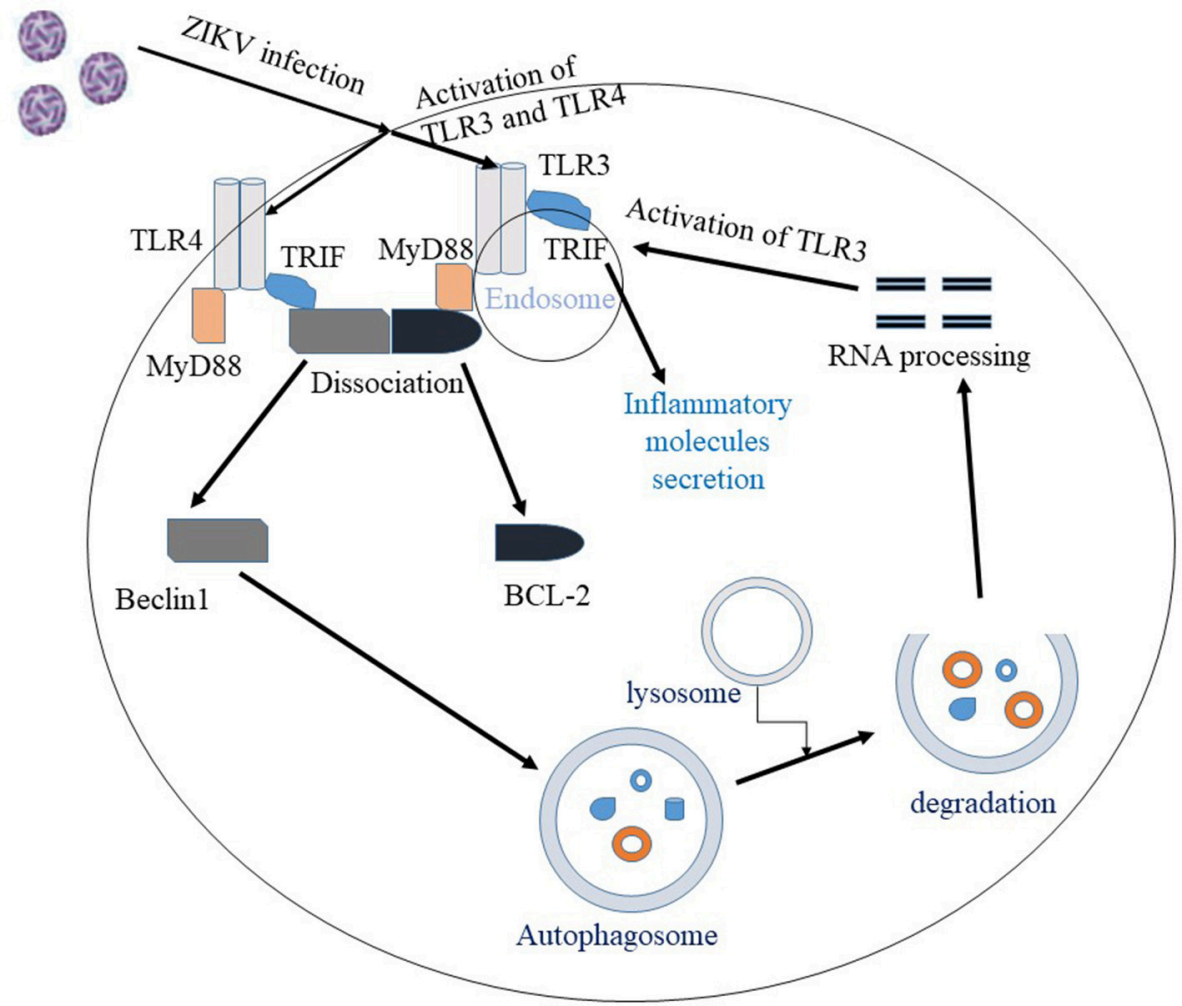
Interaction between autophagy and the TLR pathway in ZIKV infection. Once ZIKV enters the cell, TLR3 and TLR4 are activated. Activation of TLR3/4 recruit the adaptor proteins MyD88 or TRIF, which facilitate the dissociation of Beclin1 and BCL2 proteins. The unbound Beclin1 in turn initiates autophagy pathway. Autophagic degradation of the viral components leads to processing of viral RNA and the processed viral RNA may further activate TLR pathway. Overall, activation of TLR3 pathway is shown to facilitate ZIKV replication and associated pathology.

Despite the myriad of studies focusing on the molecular mechanism of ZIKV infection, there are many questions that still remain unanswered. The exact mechanisms by which the virus enters host cells and mediates brain anomalies in fetus and neurological complications in adults are still not conclusive. Evidence shows that ZIKV uses TAM and TIM receptors for viral entry and once inside the host the virus is sensed by TLR3. Activation of TLR3 leads to an antiviral response, however, exaggerated immune response mediated by hyper-activation of TLR3 may be associated with ZIKV pathogenesis. Therefore, TLR3 could be a target for therapeutic approach (20). ZIKV activates the autophagy pathway which may potentially mediate viral replication and survival within the host. Both the autophagy and the TLR3 pathways are important mediators of the innate immune response that are activated by ZIKV. Interestingly, TLR signaling pathways, in general, are reported to induce the autophagy pathway (62, 123–125). The different receptors in the TLR pathways are the initiators of both innate and adaptive immune response, while the autophagy pathway clears intracellular pathogens and facilitates antigen presentation. MyD88 and TRIF, two adaptor proteins involved in the TLR signaling pathway, can interact with the autophagy protein, Beclin1 and this interaction inhibit binding between Beclin1 and the anti-apoptotic protein, B-cell lymphoma 2 (Bcl-2). Dissociation of Beclin1 from Bcl-2 by MyD88 and TRIF induces autophagy (125), while autophagic processing of viral RNA activates TLR signaling pathway (Figure 2). In summary, ZIKV can modulate vital cellular survival and homeostatic mechanisms including the TLR, UPR, autophagy and apoptotic pathways (Figure 1), with cumulative consequences of cellular destruction and clinical manifestations. Moreover, ZIKV is capable of blocking IFN receptor (type 1) signaling to overcome the host innate immune responses.

Development of therapeutics requires precise understanding of the biology and pathogenesis of the virus. The major challenge in ZIKV research is to directly correlate viral infection with viral induced neurodevelopmental disorders in an immunocompetent animal model. Exact cellular and molecular mechanisms of ZIKV pathogenesis may not be reflective in immunocompromised mice. Therefore, development of a more appropriate animal model system is essential not only for understanding the biology of infection, but also for determining the targets for vaccines and therapies. While studying the antiviral drugs, safety in pregnant woman should also be evaluated because common antivirals and nucleoside analogs are reported to be unsafe during pregnancy, therefore alternative therapeutic approaches that target cell signaling pathways must be explored (126). Based on some of the current findings, more investigations are necessary to exploit TLR3 and the autophagy pathways as potential therapeutic targets (Figure 2). Since neurodevelopmental disorders, neuroinflammation and neurodegenerative consequences are major public health threats concerning ZIKV infection, drug delivery to central nervous system by employing targeted drug delivery approaches such as nanoparticles, nanogels, liposomes or implants need to be studied after the identification of suitable targets.

## Author contributions

NE-H conceptualization, outline and editing of the manuscript. CO preparation of the draft manuscript, preparation of figures and final assembly of manuscript. MR, JL, MM, and HB contributed by critical reading and editing of the draft manuscript. FK provided intellectual comments and editing of the manuscript. All authors read and approved the final manuscript.

### Conflict of interest statement

The authors declare that the research was conducted in the absence of any commercial or financial relationships that could be construed as a potential conflict of interest.
